# Checkpoint inhibitor is active against large cell neuroendocrine carcinoma with high tumor mutation burden

**DOI:** 10.1186/s40425-017-0281-y

**Published:** 2017-09-19

**Authors:** Victoria E. Wang, Anatoly Urisman, Lee Albacker, Siraj Ali, Vincent Miller, Rahul Aggarwal, David Jablons

**Affiliations:** 1 Department of Medicine, University of California, 1600 Divisadero Street, San Francisco, California 94115 USA; 2 Department of Pathology, University of California, San Francisco, USA; 3 Foundation Medicine, Cambridge, Massachusetts USA; 4 Department of Surgery, University of California, San Francisco, USA

**Keywords:** Large cell neuroendocrine tumor, Pembrolizumab, PD-L1, Tumor mutation burden, Immunotherapy

## Abstract

**Background:**

Large cell neuroendocrine tumor (LCNEC) of the lung is a rare and aggressive tumor similar to small cell lung cancer (SCLC). Thus, it is often treated similarly to SCLC in the front-line setting with a platinum doublet. However, treatment for patients beyond the first line remains undefined.

**Case presentation:**

We report the case of a patient with stage IB LCNEC (PD-L1 negative but positive for PD-L1 amplification and tumor mutation burden high) who progressed after adjuvant chemotherapy after surgery and subsequent therapy with an antibody drug conjugate targeting a neuroendocrine-specific cell surface marker but achieved a significant and durable response with pembrolizumab, a humanized IgG4 monoclonal anti-PD-1 antibody.

**Conclusions:**

Immunotherapy with checkpoint inhibitors is an effective treatment option for patients with metastatic LCNEC, even if PD-L1 expression is negative.

## Background

Pulmonary large cell neuroendocrine carcinoma (LCNEC) is a rare and aggressive tumor, diagnosed based on high-grade features of greater than 10 mitotic figures in 2 mm^2^ and the presence of neuroendocrine markers [1]. Its prognosis and treatment mirror that of small cell lung cancer (SCLC), with a 5-year survival rate for stage IV disease of less than 5% [1]. Compared with SCLC, however, LCNECs tend to present peripherally rather than centrally and are more likely to be early stage at diagnosis. Although there is no prospective studies guiding treatment, extrapolation based on studies performed in SCLC recommend surgical resection for early, localized disease [2]. The GFPC 0302, a phase II study, showed that the cisplatin and etoposide combination yielded similar outcome in LCNEC as in SCLC, with a median progression-free survival (PFS) of 5.2 months and overall survival (OS) of 7.7 months [3]. Recent genomic profiling efforts also revealed similarities between SCLC and LCNEC, with frequent concurrent loss of function mutations in TP53 and RB1 (~40%) [4–6]. In addition, this group harbors higher rates of MYC amplification (~15% in contrast to 6% in SCLC) [6]. The median tumor mutation burden (TMB) of LCNEC and SCLC are similar at 9.9 mutations/megabases, reflecting that both disease entities are commonly associated with the higher mutational load seen in smoking induced malignancies [6]. A second group, however, does not have TP53 and RB1 but instead possesses frequent mutations in KRAS, STK11, NOTCH1–4, and KEAP1, which are more frequently seen in non-small cell lung cancer (NSCLC) [4]. The implication for treatment and responses between these two subsets are not yet clear. Given the dismal poor overall survival rates in LCNECs, new treatment approaches for the disease are needed. Although there have been a paucity of data on PD-L1 expression in LCNEC, the recent successes of checkpoint inhibitors in relapsed SCLC after platinum chemotherapy in the phase I/II CheckMate 032 trial suggest that this might be a promising class of agents in LCNEC as well [7].

## Case Presentation

**Fig. 1:**
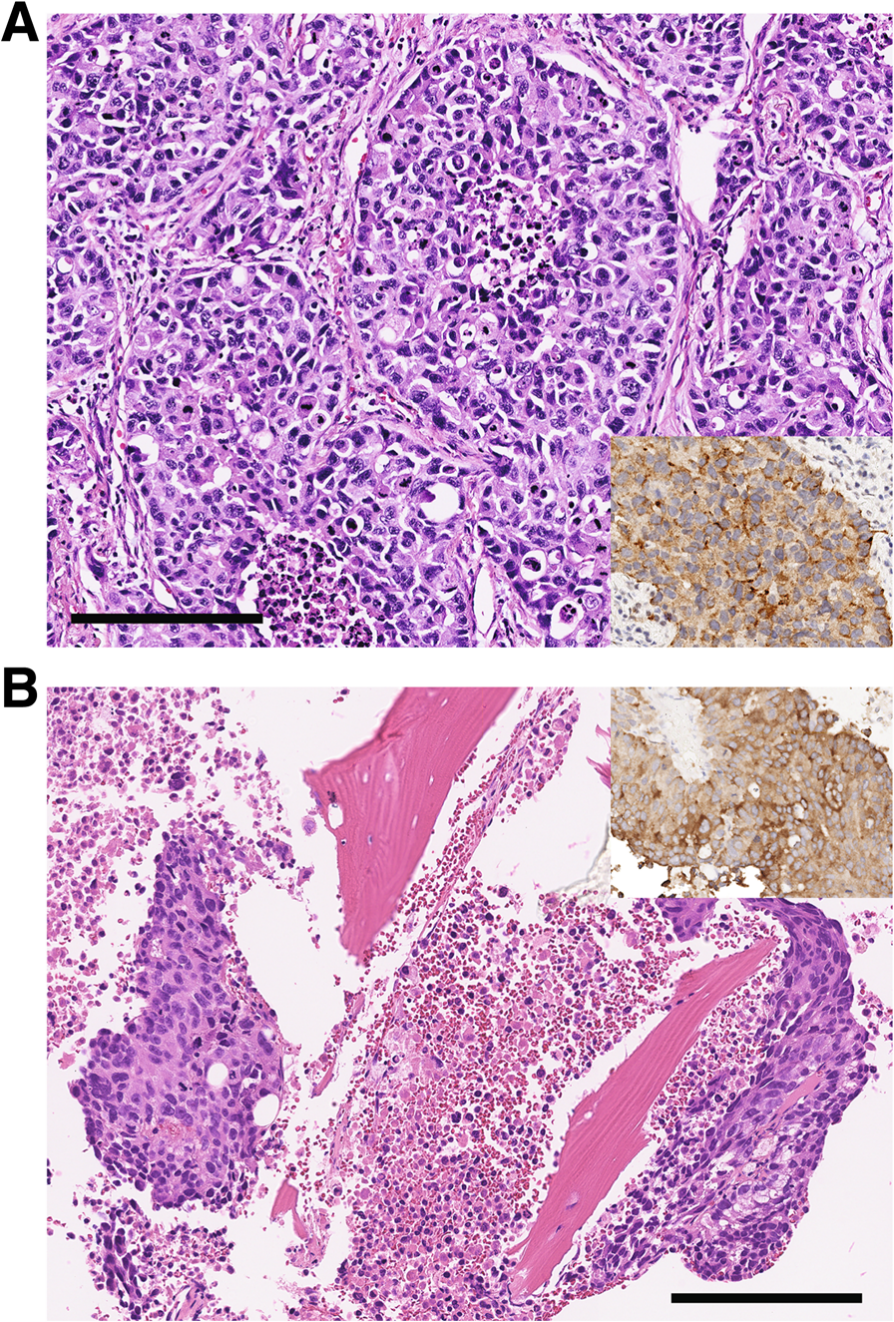
Pathologic findings. **a** Section from the initial surgical resection specimen shows a malignant neoplasm composed of solid nests of epithelioid cells with large, irregular, hyperchromatic nuclei, occasional prominent nucleoli, and abundant eosinophilic cytoplasm. There are numerous mitotic figures, and necrosis is seen in the centers of some of the tumor nests. **b** Biopsy of the iliac bone lesion shows metastatic tumor with similar histologic features. Note abundant tumor cell necrosis and fragments of bone within the biopsy. Insets in both panels demonstrate positive immunohistochemical staining for synapthophysin. [200× magnification; bar – 200 μm]

We report a case of a 64 year-old Asian man with a 40 pack-year history of smoking who presented with hemoptysis. Chest X-ray revealed a 3 cm right upper lobe nodule. CT of the chest showed a lobulated 4.2 × 4.2 × 4.8 cm lesion located in the posterior segment of the right upper lobe abutting the T3 vertebra and a 3.5 mm nodule in the right lower lobe which was deemed non-specific. CT guided biopsy initially suggested lung adenocarcinoma that was CK7 positive, TTF-1, CK5/6, 20 and p63 negative. In addition to moderate amount of centrilobar emphysema, PET-CT confirmed a FDG avid lesion spanning 4.7 × 4.9 cm with SUV of 15.8 in the right upper lobe abutting the major fissure, a 5 mm nodule in the right lower lobe too small to characterize, and greater than 1 cm level 10–14 lymph nodes with SUV of 3.3. Subsequent brain MRI was negative. A right upper lobectomy and lymph node dissection were performed. A total of 15 lymph nodes spanning levels 2, 3, 4, 7, 10, and 11 were examined and all were negative for cancer. Pathology showed a 4.7 cm poorly differentiated carcinoma with possible squamous differentiation, 1.5 cm and 1 cm from the parenchymal and bronchial margin respectively, pT2aN0M0 stage IB. Focal large blood vessel invasion was identified but without lymphatic, perineural or pleural invasion. Immunohistochemical staining was negative for TTF-1, Napsin, p40, CK5/6, and the androgen receptor but positive for CK7, synaptophysin and CD56, consistent with a large cell neuroendocrine carcinoma of pulmonary origin (Fig. 1). Greater than 20 mitosis were seen in 10 high power field. Because the tumor was high risk based on the Encore Clinical Multi-Gene Assay for Early Stage Lung Cancer, which predicts an 5-year overall survival of 44.6% [8, 9], the patient received 4 cycles of adjuvant cisplatin and docetaxel chemotherapy based on the TAX 326 study [10].

Within 4 months of completing adjuvant chemotherapy, the patient developed excruciating right hip pain. PET-CT revealed hypermetabolism at the right acetabulum and the inferolateral iliac wing (SUV 10) with cortical irregularity and “moth-eaten” erosion, concerning for metastatic infiltration. Biopsy of the right ilium showed a high-grade tumor identical to patient’s resected LCNEC. CK7, synaptophysin and CD56 were positive. At this point, the original surgical specimen was sent for molecular profiling (FoundationOne, Foundation Medicine, Cambridge). Genomic alterations identified included CD274 (PD-L1) amplification, MYC amplification, STK11 (LKB), APC, RB1, and TP53 mutations (Table 1). Tumor mutation burden was high at 24.76 mutations/megabases. Initial PD-L1 staining using the SP142 antibody was negative (Foundation Medicine, Cambridge) and independent confirmatory testing with a second 22C3 antibody (Cancer Genetics, Los Angeles) also showed <1% PD-L1 expression on the tumor cells. However, the tumor was positive for a neuroendocrine specific cell surface marker so patient received palliative radiation to the right hip and was enrolled in a phase I/II study of an antibody drug conjugate targeting this specific cell surface protein.

**Table 1:** Genomic Alterations Identified by Hybrid Capture

Gene Alteration	Loss or Gain of Function	Predictor of Response to Pembrolizumab
CD274 (PD-L1) amplification	Gain	Yes
STK11 S216F and LOH	Loss	Negative predictor
MYC amplification	Gain	No
APC E2516	Loss	No
RB1	Loss	No
TP 53 R158 L	Loss	No

**Fig. 2:**
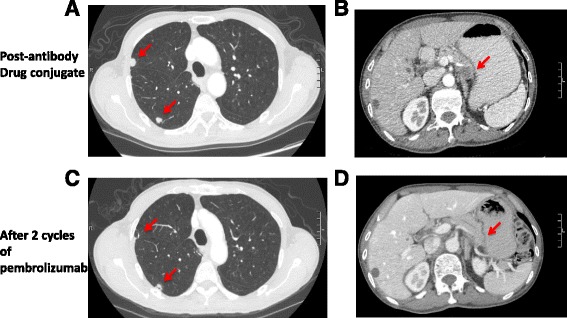
CT findings. **a** CT chest after 1 cycle of the antibody drug conjugate with new pulmonary nodules. **b** CT abdomen after 1 cycle of an experimental antibody drug conjugate against a neuroendocrine cell surface marker with a new pancreatic lesion. **c** Improvement in lung nodules after 2 cycles of pembrolizumab. **d** Resolution of the pancreatic lesion after 2 cycles of pembrolizumab

Unfortunately, after one dose of the experimental drug, patient’s disease progressed with increase in both the size and number of pulmonary nodules. Two new pancreatic lesions were also identified (Fig. 2). Patient stopped the trial and started on pembrolizumab. After 1 cycle of pembrolizumab, all visible lesions shrunk and no new lesions were seen (Fig. 2). Patient remains on pembrolizumab with continued improvement of the disease 6 months after.

## Conclusion and Discussion

LCNECs have been found in many organs, including the lung, stomach, uterus, ovary, and bladder [11–14]. Genomic profiling using a hybrid capture based assay revealed multiple alterations including CD274 (PD-L1) amplification, MYC amplification, TP53 mutation, RB1 loss, and mutations in the APC and STK11 (LKB) genes. On retrospective research review, there was loss of heterozygosity (LOH) at the STK11 locus in addition to the mutation identified, thus conferring likely a complete loss of function of STK11. TMB was high averaging 24.76 mutations per megabases. Surprisingly, PD-L1 protein expression was negative by immunohistochemistry. Genomic profiling suggested that the patient’s tumor more closely resembles the small cell type with high mutation burden, TP53 and RB1 loss, and MYC amplification. However, the presence of STK11 mutation in this patient’s tumor is more commonly associated in LCNECs that resemble NSCLC and differs from a recent genomic profiling report of 300 LCNEC where RB1 alterations was mutually exclusive to STK11 mutations (*p* < 0.001), reflecting the underlying heterogeneity and possible polyclonality of this patient’s disease [6].

PD-L1 amplification, protein levels, TMB, and STK11 mutations have all been implicated as biomarkers predictive of response to checkpoint inhibitors. While high PD-L1 amplification, protein levels, and TMB are generally associated with responses to checkpoint inhibitors, STK11 mutations are associated with decreased efficacy, at least in NSCLC [15–17]. Of these, PD-L1 amplification has been reported in only 2–4% of lung cancer based on The Cancer Genome Atlas (www.cbioportal.org) and is usually concordant with PD-L1 protein expression, although this is not the case for our patient [18, 19]. Within the SCLC literature, the incidence of PD-L1 alteration is highly controversial. Mutation was only identified in 1.6% of SCLC in the COSMIC dataset, 0/11 large cell carcinomas, and 0/54 carcinoid-endocrine tumors of the lung. Others reported PD-L1protein detection in ~70–80% of the cases (cancer.sanger.ac.uk) [20–23]. One reason for this apparent discrepancy may be attributed to the often lack of large and high quality biopsy specimen for this disease.

Here we present an index case where the a patient with LCNEC demonstrated a robust response to checkpoint inhibitors after failing several prior lines of therapy, suggesting that immunotherapy is effective in this class of disease. Interestingly, this patient had a robust response to checkpoint inhibitors despite the tumor harboring homozygous loss of function mutations in STK11 and being PD-L1 protein expression negative by immunohistochemistry. This example underscores the lack of concordance amongst current biomarkers for predicting responses to checkpoint inhibitor therapies and the need to identify more robust biomarkers. It also suggests that LCNEC may have inherently different biology compared to SCLC and adenocarcinomas of the lung. Future studies should incorporate composite biomarkers to increase the predictive power.

## Abbreviations

LCNEC: Large cell neuroendocrine carcinoma
NSCLC: Non-small cell lung cancer
OS: overall survival
PFS: Progression-free survival
SCLC: Small cell lung cancer
TMB: Tumor mutation burden
